# Preparation, assessment, and comparison of α-chitin nano-fiber films with different surface charges

**DOI:** 10.1186/s11671-015-0926-z

**Published:** 2015-05-21

**Authors:** Yan Zhang, Jie Jiang, Liang Liu, Ke Zheng, Shiyuan Yu, Yimin Fan

**Affiliations:** Jiangsu Key Lab of Biomass-based Green Fuel and Chemicals, College of Chemical Engineering, Nanjing Forestry University, Longpan Road, Nanjing, 210037 China

**Keywords:** Chitin, Nano-fiber, Films, Antimicrobial activity, Enzymatic degradation

## Abstract

Chitin nano-fibers with positive and negative charges have been, respectively, produced from partially deacetylated and 2,2,6,6-tetramethylpiperidine-1-oxyl radical (TEMPO)-mediated oxidized α-chitin. The average diameters and lengths of the TEMPO-oxidized chitin nano-fibers (TOChN) were 14 ± 4.3 and 190 ± 140 nm, respectively, and the average diameters and lengths of the partially deacetylated chitin nano-fibers (DEChN) were 6 ± 1.7 and 320 ± 105 nm, respectively. A partially deacetylated chitin nano-fiber film (DEChN-F), a TEMPO-mediated and oxidized chitin nano-fiber film (TOChN-F), and a composite film (DE-TO-ChN-F) consisting of a combination of the two were prepared by drying the dispersions at 40 °C. The DEChN-F, TOChN-F, and DE-TO-ChN-F all have similar tensile strengths of approximately 90 MPa; however, the chitosan film (Chitosan-F) had a tensile strength of approximately 30 MPa. In addition, TOChN-F and DE-TO-ChN-F have a thermal weight loss at 210 °C, and DEChN-F has a thermal weight loss at 280 °C. DEChN-F was found to have antimicrobial activity with regards to *Escherichia coli*. Finally, the chitin nano-fiber films could be slightly degraded by cellulase, which provided a novel biological performance of the chitin nano-material.

## Background

Research about natural biological materials has been ongoing for many years due to the great variety, high biosecurity, and extensive application value of these materials [1, 2]. In recent years, much attention has been paid to chitin, which has become another popular polysaccharide-based biomass for the abundant supply in nature. Chitin, occurring as the structural analog of cellulose, is mainly found in various living organisms such as outer shells of crustaceans, the cuticles of insects, and the cell walls of some fungi [3, 4]. Isolated chitin has a high crystalline structure consisting of *N*-acetylanhydroglucosamine and a few anhydroglucosamine units linked by β-(1,4)-glycoside bonds [5, 6]. Actually, chitins have been reported to be promising materials in many biomedical applications, such as tissue engineering, wound dressing, and some biocompatible devices, based upon its biocompatibility, biodegradability, and non-toxic, physiologically inert and mechanically stable properties [5, 7–10].

Native chitin crystals are primarily classified as α- and β-form allomorphs [11]. α-Chitin widely existing in crab and shrimp shells, fungal cell walls, and insect cuticle is more abundant and stable [12]. The molecular chains in α-chitin were tightly arranged antiparallely by strong intra- and inter-molecular hydrogen bonds between the amide and carbonyl groups. In contrast, β-chitin is less abundant and mainly present in squid pens and tubeworms, and it has parallel molecular chains and weaker hydrogen bonds [13, 14]. The microfibrils formed and arranged in chitin have the lateral dimensions ranged from 2.5 to 25 nm depending on their biological origins [15]. It has been known that chitin has potential to be converted into individual nano-fibers by some downsizing processes. Since nano-fibers have extremely high and active surface areas and high aspect ratios, these properties are different from the traditional materials [16]. The chitin nano-fiber also has superior mechanical performances such as a high Young’s modulus, high fraction strength, and low thermal expansion [17]. Therefore, the excellent physical and biological properties make chitin nano-fibers novel and unique candidates for the reinforcing filler in the polymer nano-composites with either of the different matrixes like poly (vinylalcohol) [18], polycaprolactone [19], chitosan [20, 21], and starch [22]. Nano-composites are a class of renewable and ecologically friendly materials, and the reinforcing fillers have at least one of its linear dimensions smaller than 100 nm [23]. The use of nano-fibers from natural polymers as reinforcing fillers, instead of the traditional inorganic materials would provide more biological or mechanical advantages including easy availability, nontoxicity, biodegradability, low density, high mechanical strength for the composite material [24].

The conventional method to prepare chitin nano-fibers or nano-whiskers is hydrolysis by strong acid and the following mechanical disintegration of the hydrolyzed solid in water. Goodrich et al. obtained α-chitin nano-whiskers from shrimp by hydrolysis with 3 M HCl and high pressure homogenization. The final nano-whiskers were 10–15 nm in diameter and 200–500 nm in length. However, the harsh treating conditions might lead to a lot of yield loss [25, 26]. Fan et al. has established two novel surface-modification methods to prepare mostly individual and highly crystallized α-chitin nano-fibers ranging from 3 to 15 nm in diameter by 2,2,6,6- tetramethylpiperidine-1-oxyl radical (TEMPO)-mediated oxidation [27] and partial deacetylation [28] with ultrasonic mechanical treatment. Selective oxidation of the C6 hydroxyl groups to carboxyl groups on the crystallite surface of α-chitin by TEMPO-mediated system could properly increase the negative charges between the fibrils. The TEMPO-mediated chitin was converted into negatively charged nano-fibers in the dispersion (TEMPO-oxidized chitin nano-fibers (TOChN)) by following homogeneous and ultrasonic treatments. Similarly, more cationic C2–NH_3_^+^ moieties were exposed on the crystallite fibrils by the partial elimination of the acetyl groups with the application of a 33 % (wt) sodium hydroxide solution at 90 °C. The positively charged nano-fibers (partially deacetylated chitin nano-fiber (DEChN)) of the partially deacetylated chitin were also obtained after the simple mechanical treatments. In both cases, electrostatic repulsion between the fibers is the key factor for the complete dispersion of the modified chitins into individual nano-fibers with negative or positive charges in water.

The chitin nano-fiber dispersions have the potential to be further processed into different forms of materials, such as cast films, hydrogels, aerogels, scaffolds, and sponges [29–33]. Especially, the materials related to chitin nano-fiber film were the most investigated. Attributed to the special bio-properties and higher mechanical strength compared with chitosan film, chitin nano-fiber film has enormous potential in various biological applications. Hence, it is significant to evaluate and characterize the biological properties of the chitin nano-fiber films. We have studied the antimicrobial and enzyme-degradable properties of both films prepared from the TEMPO-mediated and oxidized chitin nano-fiber (TOChN-F) and the partially deacetylated chitin nano-fiber (DEChN-F) with different charges in this paper. Many researchers have reported that chitosan possessed certain antimicrobial activity; however, the specific mechanism was unclear [34–37]. Ifuku et al. [20] have assessed the antifungal activity of the surface-deacetylated chitin nano-fiber/chitosan composite films, and they found that the deacetylated chitin nano-fiber film could obviously inhibit the spore germination of *Alternaria alternate* when compared with cellulose. In addition, the composite films with different nano-fiber content all showed similar inhibition with the individual chitosan film.

In the previous work, cast films with opposite charges were prepared from TEMPO-mediated and partially deacetylated α-chitin nano-fiber dispersions, and high tensile strengths and low oxygen permeabilities were found for both TOChN-F and DEChN-F [38]. In this paper, individual DEChN-F and TOChN-F were prepared and assessed firstly. Moreover, we also processed the DEChN and TOChN with opposite charges into the composite film (DE-TO-ChN-F) by the electric attraction. The further fundamental characterization of DE-TO-ChN-F, as well as DEChN-F and TOChN-F, comprising physical and biological performances, was carried out to seek new potentials of chitin nano-fibers other than the chitosans.

## Methods

### Materials

The α-chitin powder was purified from sea-crab shells with a DA (degree of N-acetylation) of 92 % that was determined by elemental analysis (Thermo Flash 2000). The chitosan powder (Sinopharm Mechanical Regent) had a DA of 10 %. Sodium hydroxide, TEMPO, sodium bromide, a sodium hypochlorite solution, and other chemicals or solvents were of laboratory grade and used without further purification. *Escherichia coli* BL21(DE3) was chosen for the antimicrobial test. Egg-white lysozyme and cellulase were purchased from Fluka and Sigma, respectively.

### Preparation and Analysis of Chitin Nano-fiber Dispersions

The DA of α-chitin decreased to 70 % after being treated with 35 % sodium hydroxide solution at 90 °C for 4 h. Then, the partially deacetylated chitin could be mechanically dispersed in distilled water at a pH 3–4 that was adjusted with acetic acid. After homogenization and ultrasonication, the partially deacetylated chitin nano-fiber dispersion with positive charges (DEChN) was achieved by removing the insoluble part. In another method, the oxidation of the C_6_-OH moieties to C_6_-COO^**−**^ on the surface of the crystalline occurred when the chitin was suspended in water containing TEMPO, sodium bromide, and sodium hypochlorite. In the oxidation system, the addition of sodium hypochlorite served as an initial oxidant to the 9 mmol g^−1^ chitin. During the reaction, the pH was maintained at a value of 10 through the addition of 0.5 M NaOH, and when the NaOH was not consumed, the oxidation ended. The carboxyl content, determined by titration with the pH-stat, was 0.7 mmol g^−1^ of chitin. Similarly, the TEMPO-oxidized chitin was also transformed into negatively charged nano-fibers (TOChN) by the following homogenization and ultrasonication. The original chitin powder, the partially deacetylated chitin (DE-Chitin) and the TEMPO-oxidized chitin (TO-Chitin) before nano-fibrillation were converted to KBr disks for FT-IR analysis. The FT-IR spectroscopy were recorded with 4 cm^−1^ on a Nicolet 6700 (Thermo Fisher). The DE-Chitin, TO-Chitin, as well as the original chitin were subjected to X-ray diffraction analysis from 5 to 50 °diffraction angle 2θ using the reflection method with the Rigaku Ultima IV at 40 kV and 30 mA. The zeta-potentials of chitin nano-fiber dispersions at 0.1 % (*w*/*w*) solid content were measured at 25 °C using a laser-Dopplerelectrophoresis-type apparatus (Zetasizer Nano ZSP). Light transmittance of the chitin nano-fiber dispersions of 0.15 % (*w*/*w*) from 300 to 700 nm was determined by an Ultrospec 2100 pro spectrometer from Amersham Biosciences, and the transmission electron microscopy (TEM) images of the nano-fibers were obtained by a JEOL electron microscope (JET 2100) at 200 kV. The rheological properties of every 0.15 % (*w*/*w*) chitin dispersion and chitosan solution were examined by a rotational rheometer (Thermo HAAKE Rheo-Stress 6000).

### Preparation of the Chitin Nano-fiber Films

α-Chitin nano-fiber films with different charges were respectively prepared from two chitin nano-fiber dispersions of DEChN and TOChN. Chitosan film was prepared from the chitosan solution. The chitosan powder can be dissolved in water by adding a little amount of acetic acid of 2 % (wt). The chitin nano-fiber dispersions or chitosan solution were poured onto plastic plates, dried at 40 °C, and then the films could be obtained by peeling them from the plates. Moreover, the chitin(+)–chitin(−) composite film (DE-TO-ChN-F) of the positively charged DEChN and negatively charged TOChN was fabricated through the layer-by-layer approach of the two nano-fiber dispersions [39]. According to the assembled mechanism, the layer-by-layer composite film consisted of alternating layers of oppositely charged chitin nano-fibers. Light transmittance of the chitin nano-fiber films with the average thickness of 0.025 mm from 300 to 700 nm was determined by an Ultrospec 2100 pro spectrometer from Amersham Biosciences.

### Determination of the Tensile Strength and the Thermostability of the Nano-fiber Films

Each nano-fiber film was tailored into several strips with lengths of 60–80 mm and diameters of 10 mm and was stretched by an electronic universal testing machine (SHIMADZU, AG-Xplus) to determine their tensile strength. In this test, the initial interval of the fixtures was 20 mm and the stretch speed was 1 mm min^−1^. A thermogravimetric analyzer (Beijing Hengjiu) was used to determine the thermostability of the chitosan and chitin nano-films. When heated from 25 to 800 °C, all of the samples would show specific weight-loss curves.

### Analysis of the Antimicrobial Properties of the Chitin Nano-fiber Films

TOChN-F, DEChN-F, and DE-TO-ChN-F were cut into squares approximately 1 cm^2^ in size, sterilized by UV irradiation, and then placed in the middle of Luria broth (LB) agar mediums. After the *E. Coli* suspension was smeared on the agar plates, the mediums were placed into the incubator at 37 °C for 14 h. Additionally, DEChN-F and DE-TO-ChN-F washed by distilled water were also assayed to take into account any influences from acetic acid.

### Enzymatic Degradability of the Chitin Nano-fiber Films

Cellulase and lysozyme were utilized to characterize the enzymatic degradability of the nano-fiber films. The degradation of the DEChN, TOChN, and DE-TO-ChN films were respectively carried out by cellulase, lysozyme, and a mixture of the two under different conditions. In the hydrolyzation of cellulase, conical flasks containing 100 mg of the chitin film fragments, 20 mL of an acetate buffer (pH 4.80), and the cellulase of 50 FPU g^−1^ were shaken at 50 °C for 24 h. In the lysozyme system, flasks containing 100 mg chitin films fragments, 20 mL of a phosphate buffer (PBS, pH 7.0), and the lysozyme of 200 U g^−1^ were shaken at 37 °C for 24 h. Finally, 100 mg film fragments were hydrolyzed by the 50 FPU g^−1^ cellulase and 200 U g^−1^ lysozyme at the same time in PBS (pH 7.0) at 40 °C for 24 h. The concentration of *N*-acetylglucosamine in the enzymatic products was detected by HPLC to evaluate the degradability of chitin nano-fiber films.

## Results and Discussion

### Preparation and Characterization of the Chitin Nano-fiber Dispersions

**Fig. 1:**
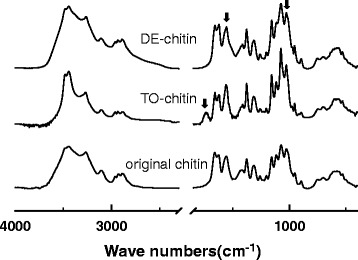
IR spectra of the original chitin powder, DE-Chitin, and TO-Chitin

**Fig. 2:**
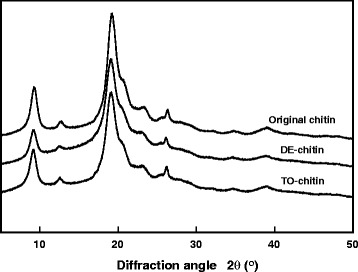
X-ray diffraction patterns of the original chitin powders, DE-Chitin, and TO-Chitin

To further verify the existence of the charged functional groups in the surface modified chitin, the chemical structures of the original, DE-Chitin and TO-Chitin, are shown in the FT-TR curves in Fig. 1. The absorption at 1030 cm^−1^ due to the C-O stretching vibration was used as an internal standard [27, 40]. After TEMPO-mediated oxidation, the TO-Chitin spectra emerged a conspicuous absorption at 1740 cm^−1^ which was caused by the carboxyl groups. As to the partial deacetylation, amide II has an absorption at 1560 cm^−1^. By comparing the absorption ratio of A1560/A1030 which was studied in the determination of the degree of deacetylation, the ratio of DE-Chitin was smaller than the original chitin [41]. It can be an evidence for the partial removal of the acetyl groups on the C2 position of chitin [27]. The X-ray diffraction patterns of the original chitin, DE-Chitin and TO-Chitin, are shown in Fig. 2; both of the two modified chitins, as well as the original chitin, had diffraction peaks at 9.5, 19.4, and 23.4 °, which were characteristic peaks belonging to the original chitin. Thus, the original crystal structure of α-chitin was not destroyed by the deacetylation and TEMPO-mediated oxidation while maintaining the original high crystallinity. Then, TO-Chitin with negative charges and DE-Chitin with positive charges homogeneously dispersed in the aqueous phase, respectively, at pH 6–8 and pH 3–4 through the electrostatic repulsion between the fibers. As shown in Table 1, zeta-potentials of the dilute dispersions certify the mechanism of the nano-fibrillation.

**Table 1:** Zeta-potentials of the TOChN and DEChN dispersions

	TOChN^a^	DEChN^b^
Zeta-potential (mV)	−43.2	+64.53

^a^TEMPO-oxidized chitin nano-fiber

^b^Partially deacetylated chitin nano-fiber

**Fig. 3:**
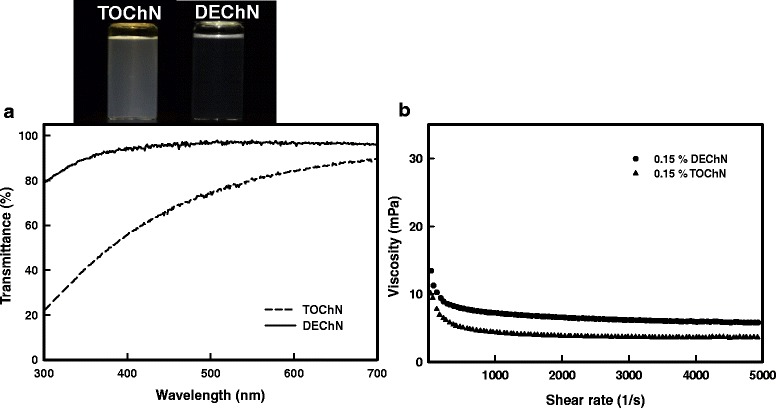
Transmittance (**a**) and viscosity (**b**) curves of DEChN and TOChN dispersions at the same concentration of 0.15 % (*w*/*w*)

**Fig. 4:**
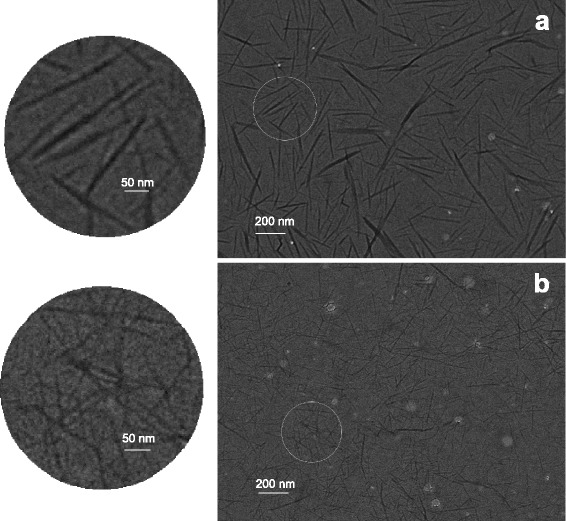
Transmission electron microscopy (TEM) images of the TOChN (**a**) and DEChN (**b**) dispersions

**Fig. 5:**
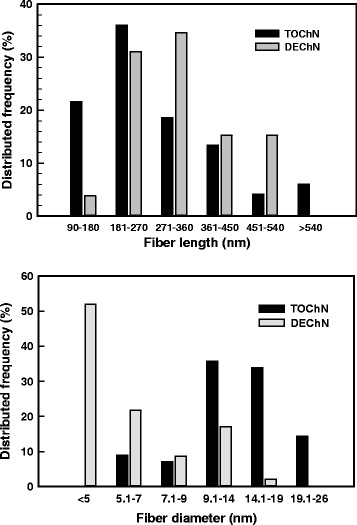
The distribution of the nano-fiber lengths and diameters in the chitin nano-dispersions

The chitin nano-fiber dispersions with certain viscosities in Fig. 3a all had an excellent transparency when compared to the distilled water. In this regard, the DEChN dispersion was more transparent than the TOChN dispersion. When sheared from 1 to 5000 1 s^−1^, the TOChN and DEChN dispersions at the same concentration of 0.15 % both showed a shear-thinning property, like the behavior of a gel (Fig. 3b). The polymer molecule chain of chitin was unwrapped along the shearing direction so that the viscosity values of the dispersions were decreased with the shear rate. Relatively, the DEChN dispersion possessed a higher viscosity between the shear range because of the longer fiber length and thinner diameter, and the TOChN dispersion possessed a lower viscosity, which was consistent with the transparency difference of the two nano-fiber types. Fiber sizes in the TEM images of the dispersions, shown in Fig. 4, obviously exhibit the fact that DEChN has a higher aspect ratio than TOChN, which is a determining factor in the viscosity difference. Fiber length and diameter distributions of the chitin nano-fibers were calculated from the TEM images and are shown in Fig. 5. The fiber length of TOChN was mainly distributed between 180 and 450 nm, and the fiber diameter was between 5 and 26 nm. The fiber diameter of DEChN was mostly concentrated between 4 and 5 nm, and the fiber length ranged between 90 and 540 nm. In conclusion, the average fiber lengths and diameters were 190 ± 140 and 14 ± 4.3 nm for TOChN and 320 ± 105 nm and 6 ± 1.7 nm for DEChN, respectively.

### Tensile Strength and Thermostability of the Chitin Nano-fiber Films

**Fig. 6:**
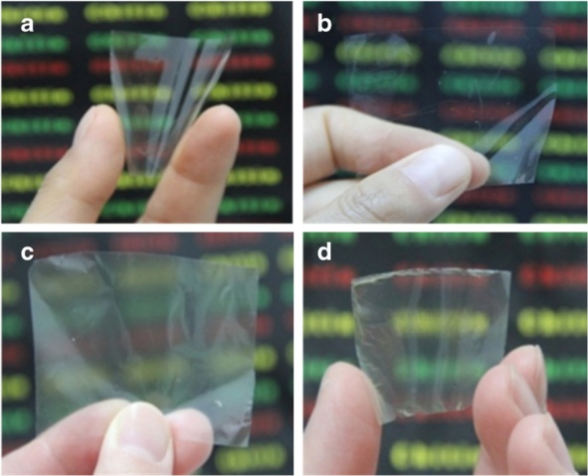
The pictures of Chitosan-F (**a**), DEChN-F (**b**), TOChN-F (**c**), and DE-TO-ChN-F (**d**)

**Fig. 7:**
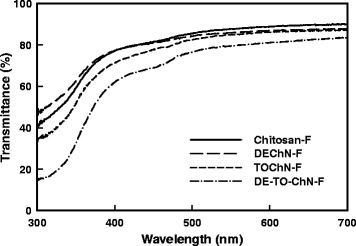
The transmittance of Chitosan-F, DEChN-F, TOChN-F, and DE-TO-ChN-F

The images of the chitosan film (Chitosan-F), DEChN-F, TOChN-F, and the chitin(+)–chitin(−) composite film (DE-TO-ChN-F) are shown in Fig. 6. Chitosan-F was studied for the comparison of the fundamental mechanical and thermal properties between the films prepared from dissolved chitosan and nano-dispersed chitin. From the appearance in the images, the Chitosan-F and DEChN-F appeared to be similar in transparency and glossiness by visual inspection, which were superior compared to the other two. By measuring the transparency of the films in Fig. 7, it is further proved that the Chitosan-F and DEChN-F simultaneously had the higher transparency than TOChN-F and DE-TO-ChN-F lowest. The high transmittance of DEChN-F mainly owe to the high aspect ratio of the DEChN.

**Fig. 8:**
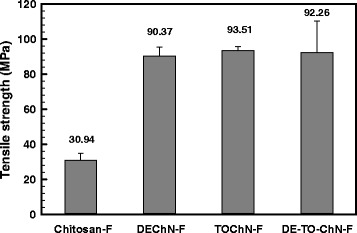
The tensile strengths of DEChN-F, TOChN-F, DE-TO-ChN-F, and Chitosan-F

As demonstrated in Fig. 8, the three types of chitin nano-fiber films possessed similar tensile strength values, which were 90.4 ± 5.2, 93.5 ± 2.2, and 92.3 ± 18.1 MPa, respectively, for DEChN-F, TOChN-F, and DE-TO-ChN-F. However, the tensile strength of Chitosan-F was only 30.9 ± 4.0 MPa, almost one third of the chitin films. The higher mechanical strength for the nano-films mainly depended upon the high degree of crystallinity of chitin and the close interaction between the nano-sized fibers. In addition, the elongations at break of Chitosan-F, DEChN-F, TOChN-F, and DE-TO-ChN-F were 5.7, 4.5, 4.3, and 7.2 %, respectively. The chitosan-F had a little stronger toughness than the two nano-fiber films with single component. It is interesting that the composite film of DEChN and TOChN showed the highest elongation at break. Therefore, the results strongly suggest that the chitin nano-fibers can be effectively utilized as reinforcements for nano-composite materials due to their high tensile strength. Combination of the chitin nano-fibers with different charges can effectively enhance the toughness of the film. It is proved to be significant to study the chitin(+)–chitin(−) nano-fiber composite materials. Chitosan could be used as a matrix to prepare novel chitosan-chitin nano-fiber composite materials with excellent mechanical strength in the future research.

**Fig. 9:**
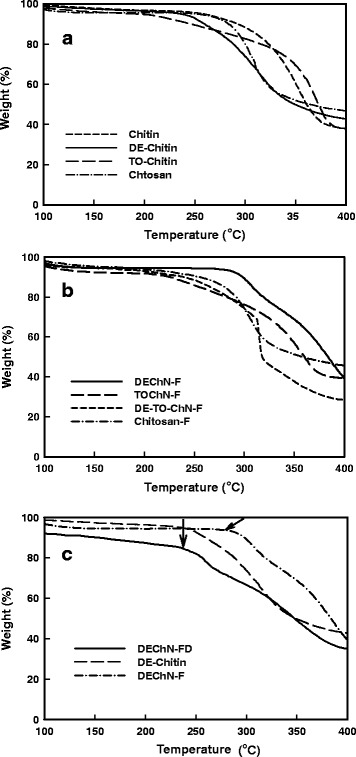
Thermogravimetric curves of **a** chitin, DE-chitin, TO-chitin and chitosan powder, **b** DEChN-F, TOChN-F, DE-TO-ChN-F, and Chitosan-F and **c** DEChN after freeze-drying (DEChN-FD)

**Fig. 10:**
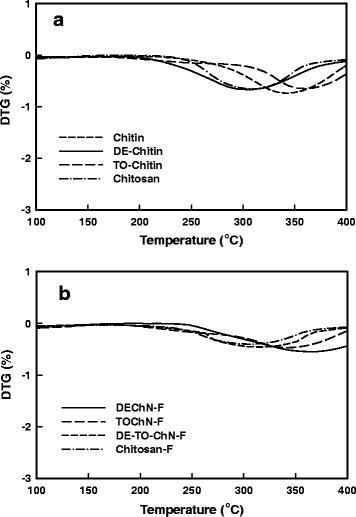
Differential thermal gravity (DTG) of **a** chitin, DE-chitin, TO-chitin, and chitosan powder and **b** DEChN-F, TOChN-F, DE-TO-ChN-F, and Chitosan-F

In this research, the thermostability of chitosan, chitin raw materials, and partially deacetylated chitin was tested to further study the physical properties. When the materials were heated from 25 to 800 °C, the thermogravimetric curves are shown in Fig. 9a; it illustrated that the initial major weight loss of chitosan and the chitin raw material occurred at approximately 250 °C, but TO-Chitin and DE-Chitin started their thermal degradation at 210 and 235 °C, respectively. It can be found that the modified chitins both had change over their initial temperatures. From the DTG analysis (Fig. 10a), the maximum degradation of chitosan and chitin raw material occurred around 300 and 350 °C, respectively. The maximum degradation temperature of TO-Chitin was close to that of chitin raw material, while for DE-Chitin, it was almost the same as that of chitosan, which indicated the different surface-modification effects. When the materials were prepared into films, the initial main degradable temperatures of Chitosan-F and TOChN-F did not change (Fig. 9b). In contrast, the thermostability of DEChN-F apparently increased to 280 °C from an initial value at 235 °C, which might be due to the thinner DEChN-F with high aspect ratio which can be arranged much tighter in the film. Meanwhile, from the DTG curve in Fig. 10b, except the DEChN-F, the maximum degradable temperature of all samples did not change when they were converted to the films. The maximum degradable temperature of DEChN-F interestingly rose near 360 °C from 300 °C of DE-Chitin. It indicated that the process of forming DEChN-F, in which the arrangement of the nano-fibers became tighter, had a greater effect on the initial degradation than on the maximum degradation. The DE-TO-ChN-F had the initial main degradable temperature approximately equal to that of TOChN-F; on the other hand, the maximum degradation temperature was around 310 °C, which might indicate more influence of TOChN on the initial degradation of the composite film. The aerogel of DEChN, achieved by freeze-drying the nano-fiber dispersion named DEChN-FD was also analyzed as depicted in Fig. 9c in order to deeply investigate whether the increase in thermostability for the DEChN-F was caused by nano-fibrillation or by the electric charges at the surface. The thermogravimetric value of DEChN-FD was equal to the value of DE-Chitin before dispersion which eliminated the possibility of nano-fibrillation. Both the DEChN-FD and DEChN-F were positively charged, but only DEChN-F changed its initial thermal degradable temperature. Therefore, the positive charges of the DEChN-F might not be the direct cause in the increase of thermogravimetric value. The reason behind and the mechanism for the change of thermostability and why TOChN-F did not exhibit this change both require further research.

### Antimicrobial Properties of the Chitin Nano-fiber Films

**Fig. 11:**
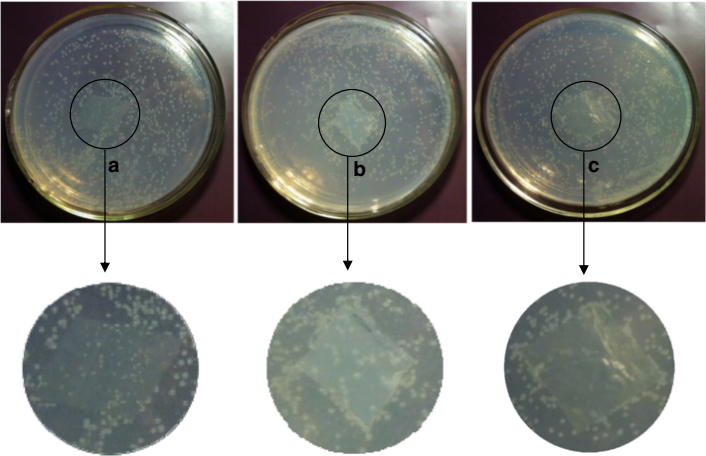
The growth of *E. coli* on DEChN-F, TOChN-F, and DE-TO-ChN-F in mediums (**a**, **b**, and **c**), respectively

The antimicrobial properties of chitosan have been well studied, and chitosan solutions were reported to have strong antibacterial activity. However, the study of Foster et.al reported a loss of this beneficial property in thin films cast from the same solutions [42]. Nevertheless, as to our knowledge, seldom has research been done on the antimicrobial properties of the self-standing thin films composed of TOChN-F, DEChN-F, or their composite film (DE-TO-ChN-F). Chitin nano-films would be preferentially utilized if they can combine both superior mechanical strength and antimicrobial activity. *E. coli* was simultaneously cultured on the two chitin nano-fiber films with different charges as well as on their composite film. Figure 11 shows that in medium A, the amount of the bacteria colonies on DEChN-F were much less and smaller than the surrounding part of the film, and the composite film in medium C displayed a similar result. On the contrary, *E. coli* grew normally on the TOChN-F as well as on the other area in medium C. It was initially demonstrated that the DEChN-F and DE-TO-ChN-F can inhibit the growth of *E. coli* to a certain degree. However, considering the DEChN were dispersed in acetic solutions, it is possible that the inhibitory effect of DEChN-F and DE-TO-ChN-F was caused by the acetic acid.

**Fig. 12:**
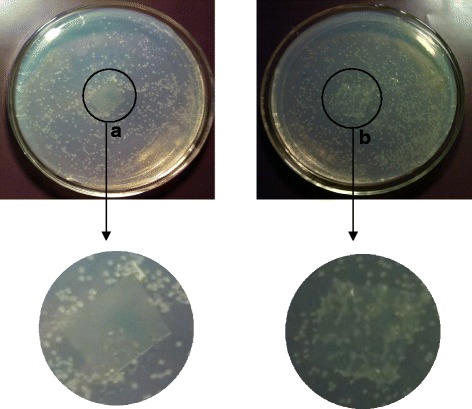
The growth of *E. coli* on DEChN-F and DE-TO-ChN-F washed to remove acetic acid in mediums (**a** and **b**)

To verify whether the antibacterial activity of DEChN and DE-TO-ChN film was due to the acetic acid, the experiment was performed on the film after it was washed in order to remove the acetic acid. Results in Fig. 12 show that the *E. coli* almost did not grow on the DEChN-F in medium A after the acetic acid was washed off. However, vigorous growth on the DE-TO-ChN-F in medium B indicated that this composite film has no antimicrobial property with regards to *E. coli* after the removal of acetic acid. Only DEChN-F revealed a definite antimicrobial property based on its inhibition to *E. coli* when the interference of acetic acid was removed. In the three films, the surface of DEChN-F was positively charged and the TOChN-F was negatively charged. The positive charges on DE-TO-ChN-F counteracted with the negative charges of TOChN-F. A hypothesis was proposed that the positive charges may have an effect on the growth of *E. coli*. Further investigation of the antimicrobial mechanism of the DEChN-F is meaningful for finding more beneficial application of the chitin nano-materials in biomedical and other fields.

### Enzymatic Degradability of the Chitin Nano-fiber Films

Egg lysozyme and cellulase were applied to study the biodegradability of the chitin nano-fiber film. Lysozyme, also known as muramidase, mainly functions to hydrolyze the β-1,4 glycosidic bonds between *N*-acetylmuramic and *N*-acetylglucosamine, while cellulase acts on the internal β-1,4 bonds of cellulose. Lysozyme commonly exists in the animal (or human) tissue, and cellulase are mainly secreted by some fungus or other organisms; the investigation of such enzymatic degradability of chitin nano-fiber film could provide a basic data for further utilization of chitin nano-fibers in the bio-field, such as the carriers for enzyme immobilization or drug delivery and so on. The study of degradability by cellulase would be also meaningful for the application of chitin nano-materials in naturally biodegradable plastics. The degree of degradability was evaluated by HPLC analysis to detect the concentration of *N*-acetylglucosamine in the solution after enzymolysis, and the results are shown in Table 2. The symbols (+) and (−) indicate whether a small amount of *N*-acetylglucosamine was detected or no *N*-acetylglucosamine was detected, respectively. After the chitin nano-fiber films were hydrolyzed by the lysozyme (200 U mg^−1^ chitin film) for 24 h, no *N*-acetylglucosamine was detected. Interestingly, a little amount of *N*-acetylglucosamine was found to exist in the cellulase hydrolyzed product, and a similar result was achieved when the films were hydrolyzed by the mixture of lysozyme and cellulase. It was proven that this degradation can be attributed to the cellulase instead of the lysozyme. The enzymatic degradability of the chitin nano-fibers is still undergoing research in order to optimize various aspects, such as the utilization of different types of enzymes, the effective dosage, and different hydrolysis conditions.

**Table 2:** The bio-enzymatic degradation of the chitin nano-fiber films

Enzymes	DEChN-F	TOChN-F	DE-TO-ChN-F
Egg lysozyme	−	−	−
Cellulase	+	+	+
Egg lysozyme + cellulase	+	+	+

## Conclusions

The chitin nano-fibers that were prepared by a partial deacetylation and a TEMPO-mediated oxidation of the chitin crystal can be further processed into films with opposite charges, as well as a composite film by electrostatic attraction. The average diameters and lengths of TOChN and DEChN were 14 ± 4.3 nm/190 ± 140 nm and 6 ± 1.7 nm/320 ± 105 nm, respectively. The DEChN has a higher degree of transparency for both the dispersion and the film due to a higher aspect ratio compared with TOChN. On the other hand, DEChN, TOChN, and their composite films all possessed tensile strengths of approximately 90 MPa, triple that of the chitosan film. In addition, only the DEChN film showed certain inhibitive effects on the growth of *E. coli*, and its thermostability increased to 280 °C from 240 °C of the original chitin material. When degraded by egg lysozyme and cellulase, the chitin films were found to only be slightly degraded by cellulase. The chitin nano-fiber film has already demonstrated a stronger mechanical strength as well as some new biological characteristics. Other properties and new forms of the material are waiting for development in order to realize novel chitin nano-fiber materials coming into our lives.

## Abbreviations

TEMPO: 2,2,6,6-tetramethylpiperidine-1-oxyl radical
TOChN: TEMPO-oxidized chitin nano-fiber
DEChN: Partially deacetylated chitin nano-fiber
TOChN-F: The film prepared from the TEMPO-oxidized chitin nano-fiber
DEChN-F: The film prepared from the partially deacetylated chitin nano-fiber
DE-TO-ChN-F: The composite film prepared from both the partially deacetylated chitin nano-fiber and the TEMPO-oxidized chitin nano-fiber
DE-Chitin: The partially deacetylated chitin powder without nano-fibrillation treatment
TO-Chitin: The TEMPO-oxidized chitin powder without nano-fibrillation treatment
Chitosan-F: The film prepared from a commercial chitosan solution
DEChN-FD: The partially deacetylated chitin nano-fiber treated by a freeze-drying of the dispersion
